# Heterozygous *SSBP1* start loss mutation co-segregates with hearing loss and the m.1555A>G mtDNA variant in a large multigenerational family

**DOI:** 10.1093/brain/awx295

**Published:** 2017-11-22

**Authors:** Peter J Kullar, Aurora Gomez-Duran, Payam A Gammage, Caterina Garone, Michal Minczuk, Zoe Golder, Janet Wilson, Julio Montoya, Sanna Häkli, Mikko Kärppä, Rita Horvath, Kari Majamaa, Patrick F Chinnery

**Affiliations:** 1 MRC-Mitochondrial Biology Unit, University of Cambridge, CB2 0XY, UK; 2 Department of Clinical Neurosciences, University of Cambridge, Cambridge, CB2 0QQ, UK; 3 Institute of Health and Society, Newcastle University, Baddiley-Clark Building, Richardson Road, Newcastle upon Tyne, NE2 4AX, UK; 4 Universidad de Zaragoza-CIBER de Enfermedades Raras (CIBERER)-Instituto de Investigación Sanitaria de Aragón, Spain; 5 Research Unit of Clinical Neuroscience, University of Oulu, Oulu, Finland and Medical Research Center Oulu, Oulu University Hospital and University of Oulu, Oulu, Finland; 6 Institute of Genetic Medicine, Newcastle University, UK

**Keywords:** mitochondrial diseases, hearing, muscle disease, neurodegeneration, genetics

## Abstract

The m.1555A>G mtDNA variant causes maternally inherited deafness, but the reasons for the highly variable clinical penetrance are not known. Exome sequencing identified a heterozygous start loss mutation in *SSBP1*, encoding the single stranded binding protein 1 (SSBP1), segregating with hearing loss in a multi-generational family transmitting m.1555A>G, associated with mtDNA depletion and multiple deletions in skeletal muscle. The *SSBP1* mutation reduced steady state SSBP1 levels leading to a perturbation of mtDNA metabolism, likely compounding the intra-mitochondrial translation defect due to m.1555A>G in a tissue-specific manner. This family demonstrates the importance of rare *trans*-acting genetic nuclear modifiers in the clinical expression of mtDNA disease.

## Teaser

The m.1555A>G mitochondrial DNA variant causes maternally inherited deafness, but shows highly variable clinical penetrance. Using exome sequencing, Kullar *et al.* identify a hypomorphic mutation in *SSBP1* that segregates with hearing loss in a family transmitting m.1555A>G, and serves as a trans-acting genetic modifier of clinical penetrance.

## Introduction

Mitochondrial dysfunction causes hearing loss in isolation and as a feature of multi-systemic mitochondrial disease. The mitochondrial variant m.1555A>G in the 12S ribosomal RNA gene *MTRNR1*, is present at an estimated 1 in 385 (0.26%) of the European population, and is necessary but not sufficient to cause maternally inherited deafness (Prezant *et al.*, 1993; Rahman *et al.*, 2012). Aminoglycosides are a recognized modifier factor but cannot account for all hearing-impaired carriers in multi-generational pedigrees, implicating additional co-segregating genetic factors (Bykhovskaya *et al.*, 1998; Guan *et al.*, 2006).

Here, we report a multi-generational family where a heterozygous start loss mutation in the core mitochondrial DNA (mtDNA) replisome protein gene, single stranded binding protein 1 (*SSBP1*), co-segregated with the m.1555A>G variant and the phenotype. This provides an explanation for the variable clinical penetrance of the disorder.

## Materials and methods

### Patients

**Figure 1:**
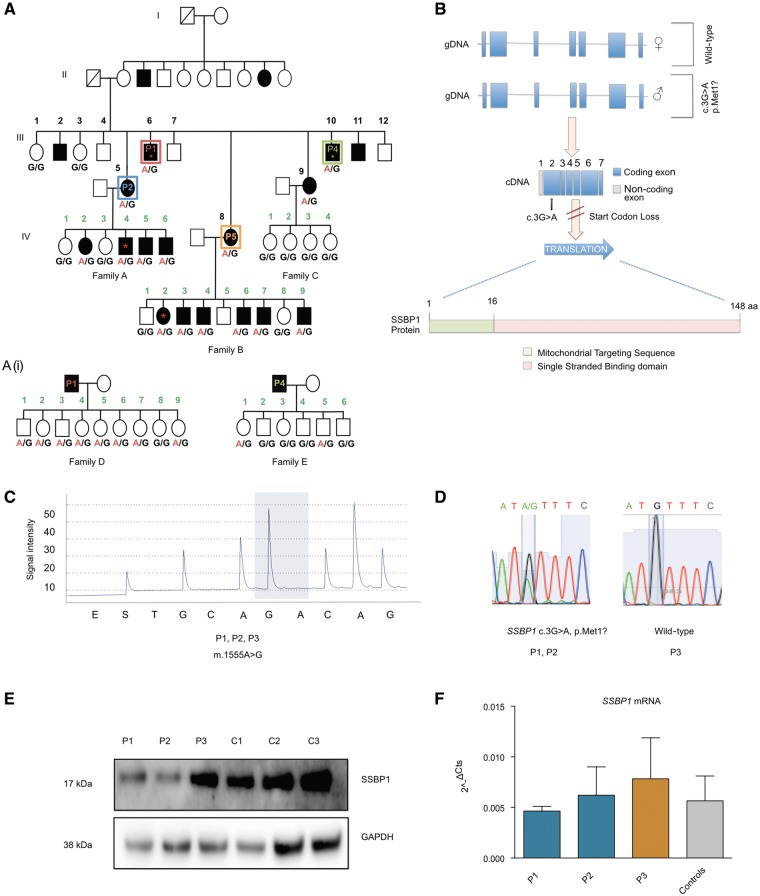
**Clinical features and genetic effect of the m.1555A>G and *SSBP1* variants.** (**A**) Family segregating m.1555A>G mtDNA variant and the c.3G>A *SSBP1* nuclear gene variant. Individuals in Generation III, 1–12 marked in black; individuals in Generation IV: Family A 1–6, Family B 1–9, Family C 1–4; marked in green. Allelic status for the c.3G>A *SSBP1* is given below each tested individual. Symbols are as follows: filled = hearing loss; unfilled = normal hearing; asterisk = exome sequenced individual; red box III-6 = Subject P1; blue box III-5 = Subject P2; green box III-10 = Subject P4; orange box III-8 = Subject P5. [**A**(**i**)] Families of III-6, P1 (*n = *9 children, Family D) and III-10, P4 (*n = *6 children, Family E). Allelic status for the c.3G>A *SSBP1* is given below each tested individual. (**B**) Schematic overview of genomic structure of *SSBP1.* Heterozygous start loss mutation, c.3G>A, abolishes primary translation start codon resulting in an effective null allele. (**C**) Representative pyrosequencing trace revealing the homoplasmic m.1555A>G mtDNA variant in Subjects P1, P2, P3. Quantified allele (G/A at m.1555) marked by blue shading. (**D**) Sequencing electropherogram from Subjects P1, P2, and P3 (unrelated m.1555A>G carrier). Subjects P1 and P2 are heterozygous for *SSBP1* c.3G>A, P3 is wild-type. (**E**) Western blot analysis reveals markedly decreased levels of SSBP1 steady state levels in Subjects P1 and P2 compared to Subject P3 and controls. Representative of three independent experiments. (**F**) *SSBP1* mRNA analysed by quantitative PCR reveals no difference between Subjects P1 and P2 compared to Subject P3 and controls (*n = *3). Data represent mean ± SD of three biological replicates.

Forty-six individuals (21 female: 25 male) carrying the m.1555A>G mtDNA variant from Northern Finland (Fig. 1A) were previously described (Hakli *et al.*, 2013). Individuals in Generation IV (nine females: 10 males) either have normal hearing (*n = *9) or sensorineural hearing loss [*n = *10, moderate high frequency (2–8 kHz) hearing loss *n = *3, moderate pan-frequency (0.25–8 kHz) hearing loss *n = *6, profound pan-frequency hearing loss *n = *1]. The mean age of hearing loss diagnosis was 3.7 years (range 1.6–5.4 years). There was no history of aminoglycoside usage. Neurological examination of all individuals was otherwise normal. There was no clinical evidence of either proximal or distal myopathy. DNA was available from 25 individuals from Generation III and IV. We subsequently also identified the children of two fathers in Generation III [Subject III-6 (P1), father in Family D, *n = *9 children; and Subject III-10 (P4), father in Family E, *n = *6 children; mean age of children* = *10.3 years (range 1–19 years); Fig. 1A(i)]. Fibroblast cell lines were established from Subjects III-6 (P1) and III-5 (P2). We also studied fibroblasts and DNA from an unrelated individual carrying m.1555A>G (Subject P3). Skeletal muscle biopsy was obtained from Subjects III-10 (P4, age 38 years) and III-8 (P5, age 41 years).

### Molecular genetics

Exome sequencing, variant calling and filtering to isolate heterozygous candidate variants with a minor allele frequency (MAF) <1% were performed as previously described (Keogh *et al.*, 2015). Sanger sequencing was performed to confirm segregation of the *SSBP1* variant. Pyrosequencing (Qiagen) was undertaken for allelic quantification of m.1555A>G. Long-range PCR was used to detect mtDNA deletions using primers (D1R m.19-1 and D2F m.1650-1671) amplifying a product encompassing almost the complete mtDNA. mtDNA copy number was determined using real time PCR TaqMan® assays targeting *MT-ND1* or *MT-CO3* and the nuclear genes, *B2M* or *RNaseP* as described (Grady *et al.*, 2014). Total DNA was prepared by phenol-chloroform extraction and precipitation for 7S DNA analysis, then linearized by PvuII digestion before electrophoresis on 0.7% agarose gels and Southern hybridization (Kornblum *et al.*, 2013). RNA was extracted from fibroblast cell lines using RNeasy® Mini Kit (Qiagen) and cDNA was synthesized using High-Capacity cDNA Reverse Transcription Kit (Applied Biosystems). Quantification of gene expression was performed using the TaqMan® Gene Expression Assay using transcript specific primers for *SSBP1* and *MT-CYB* with normalization to *GAPDH*.

### Biochemical analysis of the oxidative phosphorylation system

Protein extract from fibroblasts was separated by electrophoresis on a 4–12% polyacrylamide gel (NuPAGE). Immunoblotting was performed as described using antibodies to SSBP1, (12212-1-AP, Proteintech), mitochondrially encoded cytochrome *c* oxidase 1 (MT-CO1, ab14705, Abcam), mitochondrially encoded cytochrome *c* oxidase 2 (MT-CO2, 12C4F12, Abcam), succinate dehydrogenase complex flavoprotein subunit A (SDHA, ab14715, Abcam), ATP synthase subunit alpha (ATP5A, MS604, Mitosciences), translocase of outer membrane 20 (TOMM20, ab56783, Abcam) and glyceraldehyde-3-phosphate dehydrogenase (GAPDH, sc-25778, Santa-Cruz) (Pfeffer *et al.*, 2014). Cellular oxygen consumption rate (OCR) was assayed using the Seahorse XF96 Extracellular Flux Analyser with sequential addition of oligomycin (1 μM), carbonyl cyanide 4-(trifluoromethoxy)phenylhydrazone (FCCP, 1 μM) and rotenone/antimycin (1 μM) to measure basal and maximal respiration. Cell growth was assessed by growing fibroblast cells with high glucose medium versus glucose-free medium supplemented with 5 mM galactose using IncuCyte® Live Cell imager, Essen Bioscience.

### Muscle histochemistry

Haematoxylin and eosin, cytochrome *c* oxidase (COX), succinate dehydrogenase (SDH) and sequential COX-SDH staining was performed on sectioned skeletal muscle (10 μm transverse sections) as previously described (Taylor *et al.*, 2004).

### Intra-mitochondrial translation assay

Metabolic labelling of mitochondrial proteins was performed as previously described (Van Haute *et al.*, 2016). Loading was assessed by western blotting for TOMM20.

## Results

### Sequencing analysis

Pyrosequencing of leucocyte DNA confirmed all subjects were homoplasmic for m.1555A>G (Fig. 1C). Exome sequencing was performed on four affected individuals [Subjects III-6 (P1), III-10 (P4), IV-4 (Family A) and IV-2 (Family B)]. Average coverage ≥10 fold was obtained for 93.2% of bases (range 89.6–94.9%). After filtering, we identified a heterozygous c.3G>A variant in *SSBP1* (NM_001256510:exon2:c.G3A:p.M1?) that abolishes the primary start codon (Fig. 1B). The variant is found in 16 of 267 458 sequenced alleles in the gnomAD database (http://gnomad.broadinstitute.org). All of the variants were found in Finnish individuals (16 of 25 294 sequenced alleles), possibly indicating a founder effect within the Finnish population. The variant is predicted to be pathogenic by MutationTaster = 1, SIFT = 0 and LRT = 0 (Chun and Fay, 2009; Kumar *et al.*, 2009; Schwarz *et al.*, 2010). Sanger sequencing demonstrated the variant segregated with the hearing loss in all sequenced individuals in Generation III and IV (Fig. 1A and D). Sanger sequencing revealed children in Family D (*n = *8/9, 89%) and Family E (*n = *2/6, 33%) carried the c.3G>A *SSBP1* but all carried wild-type m.1555A [Fig. 1A(i)]. Subject P3 and a cohort of nine unrelated hearing-impaired m.1555A>G carriers had a wild-type *SSBP1* sequence.

### SSBP1 protein levels

Western blotting revealed decreased steady-state SSBP1 levels in fibroblasts from Subjects P1 and P2 compared to the m.1555A>G cell line (Subject P3) and controls (Subjects C1–3) (Fig. 1E). No significant difference in the mRNA expression of *SSBP1* was found between patient and control fibroblasts (Fig. 1F), consistent with the variant abolishing *SSBP1* translation without an effect on transcription.

### Muscle and fibroblast mtDNA analysis

**Figure 2:**
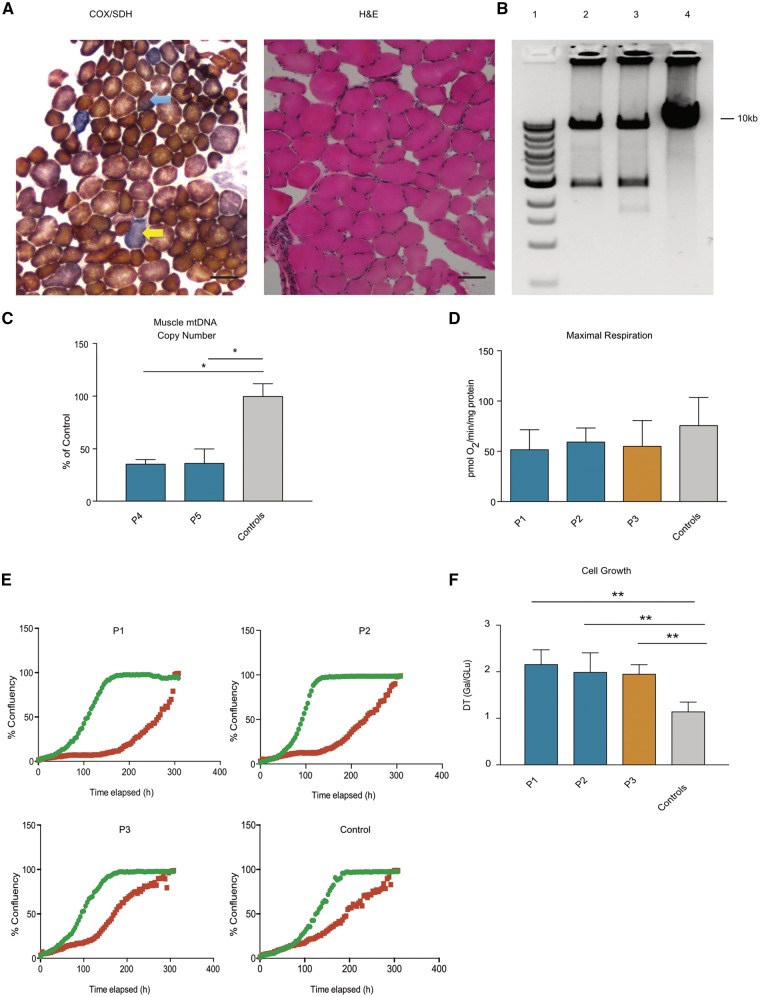
**Muscle histochemistry and mtDNA copy number, analysis of cellular respiration and cell growth.** (**A**) Muscle histochemistry of Patient P5. Sequential cytochrome *c* oxidase / succinate dehydrogenase (COX/SDH) staining reveals the presence of COX negative fibres (yellow arrow) and COX intermediate fibres (blue arrow). Haematoxylin and eosin (H&E) staining reveals fibre size variation within normal limits. (**B**) Long range PCR of muscle DNA shows evidence of multiple mtDNA deletions. Lane 1 = DNA size marker; Lane 2 = Subject P4; Lane 3 = Subject P5; Lane 4 = Control. (**C**) mtDNA copy number analysis as determined by qPCR targeting *MT-CO3* and *RNaseP*, Subject P4 = 35.8 ± 4, Subject P5 = 36.5 ± 13.3% of controls (*n = *2). Data represent mean ± SD of two independent determinations, *P < 0.05, one-way ANOVA with *post hoc* Tukey test. (**D**) Measurement of cellular oxygen consumption rate by Seahorse XF96 Extracellular Flux Analyser reveals a trend towards lower maximal respiration in Subjects P1, P2, P3 compared to controls (*n = *3). Data represent mean ± SD of four independent experiments. (**E**) Cellular growth curve analysis of Subejects P1, P2, P3 and control on glucose (green line) and glucose-free media supplemented with 5 mM galactose (red line). Graphs represent cell confluency (%) versus time elapsed (h). Data representative of three independent experiments. (**F**) Quantification of doubling time (DT) of Subjects P1, P2, P3 and controls (*n = *2) in galactose media normalized to growth on glucose media. Data represent mean doubling time ± SD of three independent experiments, **P ≤ 0.01, one-way ANOVA with *post hoc* Tukey test.

**Figure 3:**
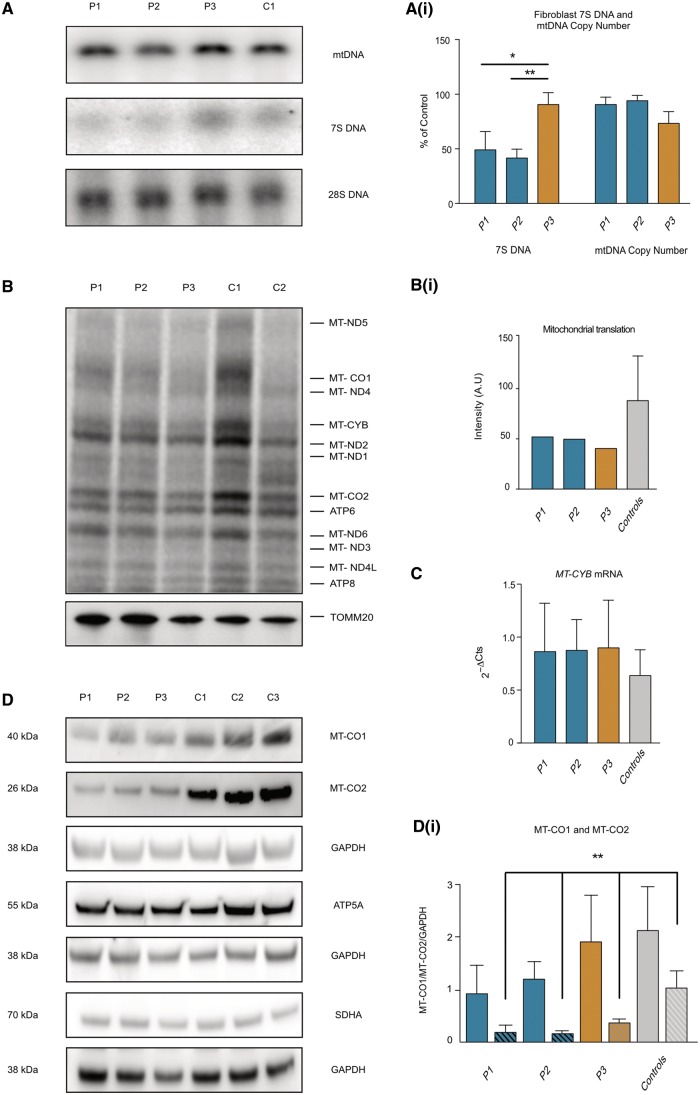
**7S DNA, mtDNA copy number, intra-mitochondrial translation and mitochondrial gene expression.** (**A**) Southern blot analysis of mtDNA copy number and 7S DNA levels in Subjects P1, P2, P3 and control fibroblasts. *PvuII* digested DNA was hybridized with a radiolabelled probe A (mtDNA position 16270–16292 and 389–369), which detects both genomic mtDNA and 7S. 28S was used as a loading control. [**A**(**i**)] Quantification of mtDNA and 7S DNA levels in Subjects P1, P2, P3 and control. 7S DNA levels normalized to genomic mtDNA levels, Subject P1 = 49.1 ± 16.7, Subject P2 = 41.6 ± 8.1, Subject P3 = 90.6 ± 10.8% of control. Mean ± SD of three independent experiments *P < 0.05, **P < 0.01 one-way ANOVA with *post hoc* Tukey test. Blood and fibroblast mtDNA levels assayed by qPCR targeting *MT-ND1* and *B2M* detects no difference between Subjects P1, P2, P3 and controls (*n = *3). Data represent mean ± SD of three biological replicates (blood data not shown). (**B**) Intra-mitochondrial protein translation analysis by incorporation of 35S methionine reveals a clear reduction of mitochondrial protein synthesis in Subjects P1, P2 and P3 compared to controls. Representative of three independent experiments. The characteristic bands of mitochondrial encoded proteins are annotated (MT-ND1, MT-ND2, MT-ND3, MT-ND4L, MT-ND5, MT-ND6: NADH dehydrogenase subunit 1,2,3,4L,5,6; MT-CO1, MT-CO2: mitochondrially encoded cytochrome *c* oxidase, I, II; MT-CYB: mitochondrially encoded cytochrome *b*; ATP6, ATP8: mitochondrially encoded ATP synthase 6,8). Loading determined by TOMM20 western blot. [**B**(**i**)] Quantification of band intensities from **B**. (**C**) Quantitative PCR analysis reveals no difference in *MT-CYB* mRNA levels between Subjects P1, P2, P3 and controls. Data represent mean ± SD of three biological replicates. (**D**) Representative western blot analysis of fibroblast cell lysates from Subjects P1, P2, P3 and controls reveals reduced steady state levels of MT-CO1 and MT-CO2 without change in nuclear encoded mitochondrial proteins ATP5A (ATP synthase subunit alpha) and SDHA (succinate dehydrogenase complex flavoprotein subunit A). [**D**(**i**)] Quantification of MT-CO1 (solid bars) and MT-CO2 (hashed bars) reveals a trend towards reduced MT-CO1 in Subjects P1 and P2 and significantly reduced MT-CO2 in Subjects P1, P2 and P3 compared to controls. Data represent mean ± SD of three independent experiments, **P < 0.01, one-way ANOVA with *post hoc* Tukey test.

Haematoxylin and eosin staining of the muscle biopsy from Subject P5 was unremarkable whereas sequential COX-SDH staining revealed evidence of respiratory chain deficiency with a low number of COX-deficient and COX-intermediate fibres (5/174, 3.4%, Fig. 2A) (Murphy *et al.*, 2012). Long-range PCR of muscle DNA from Subjects P4 and P5 showed the presence of multiple mtDNA deletions (Fig. 2B), and muscle mtDNA copy number was reduced by ∼60% in Subjects P4 and P5 compared to controls (Fig. 2C), both suggestive of disordered mtDNA maintenance. We did not detect mtDNA copy number changes in patient blood or fibroblast DNA [Fig. 3A(i)], thus demonstrating tissue specificity.

### Respiration and growth analysis

Measurement of cellular OCR in patient and control fibroblasts revealed no significant difference in basal OCR but a trend of lower maximal respiration in Subjects P1, P2 and P3 compared to controls (Fig. 2D). Subject P1, P2 and P3 cells had significantly greater doubling times than controls when galactose was used as a carbon source (Fig. 2E and F).

### 7S DNA analysis

Mammalian mtDNA molecules contain a triple-stranded region (D-loop) found in the major non-coding of many mitochondrial genomes, formed by stable incorporation of a third, short DNA strand known as 7S DNA. The exact function of 7S DNA is unknown; however, it has been proposed to play a role in replication as an intermediate of prematurely-terminated heavy (H-) strand synthesis and moreover, perturbations in the steady-state levels of 7S DNA have been observed in a mtDNA maintenance disorder (Kornblum *et al.*, 2013; Nicholls and Minczuk, 2014). Previous work has shown that SSBP1 is required for mtDNA replication and regulates the mtDNA D-loop by modulating the synthesis of 7S DNA (Ruhanen *et al.*, 2010). We analysed the abundance of 7S DNA relative to genome length mtDNA molecules in patient and control fibroblasts. Subjects P1 and P2 had significantly reduced 7S DNA abundance compared to Subject P3 and controls. There was no difference in the level of full-length mtDNA relative to nuclear 28S DNA in Southern blots, confirming the lack of difference in mtDNA copy number in Subject P1, P2 and P3 fibroblasts compared to controls [Fig. 3A and A(i)].

### Mitochondrial mtDNA transcription and protein synthesis in fibroblasts

Measurement of *de novo* intra-mitochondrial protein translation by incorporation of ^35^S radiolabelled methionine revealed markedly reduced global mitochondrial protein synthesis in Subject P1, P2 and P3 cells compared to controls [Fig. 3B and B(i)]. Reduction in protein synthesis in patient cells was accompanied by a reduction in the steady state levels of mtDNA-encoded complex IV subunits, MT-CO1 in Subjects P1 and P2 and MT-CO2 in Subjects P1, P2 and P3 [Fig. 3D and D(i)]. There was no difference in steady state levels of nuclear encoded mitochondrial proteins ATP5A and SDHA (Fig. 3D) or in *MT-CYB* mRNA expression (Fig. 3C). Together these findings reveal a defect in mitochondrial translation mediated by m.1555A>G that was no more severe in the presence of the *SSBP1* variant.

## Discussion

Here we describe a heterozygous start loss mutation in *SSBP1* co-segregating with hearing loss in a maternal pedigree transmitting the m.1555A>G variant. The background frequency of m.1555A>G in northern Finland has been estimated to be significantly less than in other European populations (0.0047% versus 0.26%, Fisher’s exact *P* ≤ 0.001) reducing the likelihood that our observations are the result of a chance finding (Lehtonen *et al.*, 2000; Rahman *et al.*, 2012).

The *SSBP1* mutation reduced SSBP1 levels, decreased 7S DNA in fibroblasts, and was associated with multiple deletions of mtDNA and mtDNA depletion in skeletal muscle. Fibroblasts from these patients also showed reduced intra-mitochondrial protein synthesis in keeping with the co-existing m.1555A>G variant, leading to reduced proliferation rates under conditions of forced mitochondrial respiration (Guan *et al.*, 1996).

Patients with defects in *POLG*, another mitochondrial maintenance gene encoding the mitochondrial DNA polymerase, polγ, are thought to accumulate mtDNA deletions by replication stalling at homopolymeric tracts. It has been proposed that SSBP1 reduces arrests within these tracts, and thus suppresses mtDNA deletion formation (Mikhailov and Bogenhagen, 1996). Given that SSBP1 also coats the H-strand during replication, low SSBP1 levels in our patients may increase mtDNA replication stalling and non-specific replication initiation, compromising replication fidelity leading to tissue-specific mtDNA deletion and depletion (Miralles Fuste *et al.*, 2014). The observed reduction in 7S DNA in Subjects P1 and P2 fibroblasts is in keeping with dysfunctional mtDNA replication. The absence of detectable mtDNA deletions in fibroblasts is well recognized in mtDNA maintenance disorders (Stewart *et al.*, 2011), probably because the deletions are rapidly lost in rapidly dividing cells.

Of note, we specifically searched for a common variant in *TRMU* (c.28G>T p.A10S, gnomAD frequency = 0.097), that has previously been suggested to modify the phenotype in a subset of m.1555A>G carriers (Guan *et al.*, 2006; Meng *et al.*, 2017). Reflecting this allele frequency, the variant was found in both hearing impaired (3/8, 37.5%) and normal hearing (2/5, 40%) individuals in the family, so cannot account for the phenotype in our patients. Similarly, the *SSBP1* variant is unlikely to be pathogenic in isolation given that 10/15 (67%), of the children in Families D and E carry the *SSBP1* variant in conjunction with wild-type m.1555A and all have normal hearing. In addition, the heterozygous knockout mouse Ssbp1^tm1a(KOMP)Wtsi^ displays only a mild phenotype, with hearing no different to wild-type littermates (Brown and Moore, 2012).

Our findings are therefore consistent with an additive effect of the *SSBP1* mutation and m.1555A>G, with a combined effect on mtDNA translation and mtDNA maintenance causing a tissue-specific phenotype. Although there is both microscopic and molecular evidence of muscle disease, the patients do not display overt clinical signs of myopathy. The clinical features in these individuals were limited solely to the auditory system.

Taken together, these data suggest rare trans-acting alleles are important modifiers of the m.1555A>G phenotype; this should be taken into consideration for appropriate genetic counselling of carriers and their families.

## Funding

P.J.K. is a Wellcome Trust Clinical Research Training Fellow (101700/A/13/Z). P.F.C. is a Wellcome Trust Senior Fellow in Clinical Science (101876/Z/13/Z), and a UK NIHR Senior Investigator, who receives support from the Medical Research Council Mitochondrial Biology Unit (MC_UP_1501/2), the Wellcome Trust Centre for Mitochondrial Research (096919Z/11/Z), the Medical Research Council (UK) Centre for Translational Muscle Disease research (G0601943), EU FP7 TIRCON, and the National Institute for Health Research (NIHR) Biomedical Research Centre based at Cambridge University Hospitals NHS Foundation Trust and the University of Cambridge. The views expressed are those of the author(s) and not necessarily those of the NHS, the NIHR or the Department of Health. C.G. is supported by the European Commission under “Marie Skłodowska-Curie Actions”, Individual Fellowship – Reintegration Panel (Mitobiopath-705560). J.M. receives funding from the Instituto de Salud Carlos III (PI14/00005 and PI14/00070); Departamento de Ciencia, Tecnología y Universidad del Gobierno de Aragón (Grupos Consolidados B33) and FEDER Funding Program from the European Union; and Asociación de Enfermos de Patología Mitocondrial (AEPMI). P.A.G. and M.M. are supported by core funding from Medical Research Council (UK) (MC_U105697135).
